# The Effect of FcRn Binding on Ocular Disposition of Monoclonal Antibodies

**DOI:** 10.3390/antib15020027

**Published:** 2026-03-25

**Authors:** Sanika Naware, Saurav Kulkarni, Sahil Salvi, Dhvani Patel, Dhaval K. Shah

**Affiliations:** Department of Pharmaceutical Sciences, School of Pharmacy and Pharmaceutical Sciences, The State University of New York at Buffalo, Buffalo, NY 14214-8033, USA; snaware@buffalo.edu (S.N.); spkulkar@buffalo.edu (S.K.); sahilsal@buffalo.edu (S.S.); dhvanipr@buffalo.edu (D.P.)

**Keywords:** monoclonal antibodies, neonatal Fc receptor, ocular pharmacokinetics, tear fluid, biodistribution, rabbit

## Abstract

**Background/Objectives**: The neonatal Fc receptor (FcRn) plays a crucial role in extending the systemic half-life of monoclonal antibodies (mAbs), but its influence on ocular distribution remains incompletely understood. This study investigated the impact of FcRn on the ocular disposition of mAbs following systemic administration in rabbits. **Methods**: New Zealand White rabbits received a single intravenous dose (1 mg/kg) of either wild-type trastuzumab (TS-WT) or its FcRn non-binding variant (IHH). Plasma and ocular tissues (retina, iris–ciliary body, vitreous humor, aqueous humor, cornea, conjunctiva, and tears) were collected at terminal time points up to 336 h for TS-WT and 168 h for IHH. Antibody concentrations were quantified using a validated sandwich ELISA. Pharmacokinetic parameters and antibody biodistribution coefficients (ABC) were calculated to assess the FcRn-mediated effects on ocular distribution. **Results**: TS-WT demonstrated 2-fold higher systemic exposure compared to IHH. The iris–ciliary body exhibited the highest absolute exposure for both antibodies, with TS-WT showing significantly higher accumulation (ABC_0–168h_: 14.95% vs. 8.89%). Retinal distribution remained comparable between antibodies (5.96% vs. 5.51%). Both antibodies were detectable in tears, with ABC value of ~4% reported for TS-WT. TS-WT also demonstrated markedly increased distribution in vitreous humor and tear fluid (3.5- and 5.5-fold higher ABC values, respectively) compared to IHH. The cornea (5.76% vs. 5.57%) and conjunctiva (7.71% vs. 7.21%) showed comparable relative distribution between TS-WT and IHH, while aqueous humor showed minimal differences (0.44% vs. 0.52%). **Conclusions**: This investigation reveals distinct tissue-specific patterns of FcRn-mediated mAb distribution within the eye. FcRn binding significantly enhanced antibody distribution in ocular tissues, such as the iris–ciliary body, and tears, with less pronounced effects on the retina, cornea, conjunctiva and aqueous humor. These findings provide mechanistic insights for optimizing mAb-based therapeutics for ocular disease and understanding the ocular toxicity of mAb-based therapeutics, such as antibody–drug conjugates.

## 1. Introduction

Protein therapeutics, especially monoclonal antibodies (mAbs), have emerged as a powerful class of therapeutics, owing to their high target specificity and extended systemic half-lives. In fact, most FDA-approved therapies for ocular disorders such as diabetic retinopathy (DR), age-related macular degeneration (AMD), and retinal vein occlusion (RVO) are protein therapeutics, including aflibercept (Eylea), ranibizumab (Lucentis), brolucizumab (Beovu), faricimab (Vabysmo), and pegaptanib sodium (Macugen). Despite their therapeutic promise, understanding the pharmacokinetics (PK) of mAbs in ocular tissues remains a challenge due to the anatomical and physiological barriers of the eye, involving static barriers like the blood–retinal barrier (BRB), blood–aqueous barrier (BAB), cornea and sclera; dynamic barriers such as the lymphatic network, tear turnover, and nasolacrimal drainage impede the movement of the drug into the ocular tissues [1]. Among the various molecular determinants that influence mAb disposition—such as molecular size, charge, hydrophobicity, and target binding—the neonatal Fc receptor (FcRn) has received increasing attention for its role in regulating the systemic half-life and tissue distribution of mAbs, including within the eye.

FcRn is expressed in a variety of tissues, including vascular endothelium and epithelial cells, where it functions to bind the Fc region of IgG at acidic pH and protect it from lysosomal degradation, facilitating IgG recycling and transcytosis across cellular barriers [2]. While the role of FcRn in prolonging the systemic half-life of mAbs is well-established, its influence on the ocular disposition of mAbs is not yet fully understood. The emerging evidence suggests that FcRn may mediate the active transport of IgG across ocular barriers such as the blood–retinal barrier (BRB) and blood–aqueous barrier (BAB), thereby affecting local exposure and systemic clearance following intraocular or systemic administration [3].

In this study, we investigated the impact of FcRn on the ocular disposition of mAbs, using rabbits as the preclinical model species. Rabbits are one of the most widely used species in ophthalmic research due to their relatively large globe size, which is comparable to that of humans; ease of handling; cost-effectiveness; rapid breeding; and longer lifespan. These characteristics make them particularly suitable for studying age-related ocular diseases such as glaucoma and AMD, as well as evaluating sustained release ocular drug delivery systems like intraocular lenses and implants [4]. Rabbits were administered with intravenous injections of either trastuzumab or its FcRn non-binding variant, which carries Fc region mutations (IHH mutated) that abrogate FcRn binding [5]. Trastuzumab is a humanized IgG1 monoclonal antibody that targets human epidermal growth factor receptor 2 (HER2) but does not cross-react with rabbit HER2, and thus it serves as a non-targeting control [6]. Plasma and ocular tissues, including the retina, iris–ciliary body, aqueous humor, vitreous humor, cornea, conjunctiva, lens and tears were collected at different terminal time points to evaluate the ocular disposition of both the mAbs. By comparing the ocular and systemic PK profiles of wild-type and FcRn non-binding mAb, this work aims to provide mechanistic insights into the role of FcRn governing ocular disposition of mAbs.

Our findings will have implications for the rational design and optimization of mAb-based therapeutics for ocular diseases, particularly those relying on systemic administration or Fc engineering strategies aimed at modulating FcRn interactions.

## 2. Materials and Methods

### 2.1. Antibody Production and Purification

Wild-type trastuzumab (TS-WT) and FcRn non-binding (IHH) trastuzumab was produced using an established CHO cell expression system that was previously developed in the laboratory. CHO cells comprising the IGK-FRT-based expression vector were cultured in EX-CELL^®^ Advanced CHO Fed-batch Medium (Sigma-Aldrich, St. Louis, MO, USA; Cat# 24366C) for three weeks. Following culture, the supernatant was clarified by centrifugation at 10,000× *g* for 15 min and subsequently filtered. The antibody was initially purified using a HiTrap™ Protein G HP column (GE Healthcare, Chicago, IL, USA; Cat# 12490017), followed by further purification using a Foresight™ CHT™ Type II column (Bio-Rad, Hercules, CA, USA; Cat# 7324756) on the NGC chromatography system (Bio-Rad, Hercules, CA, USA). The final product was buffer-exchanged into phosphate-buffered saline (1× PBS) and stored at 4 °C. The identity and purity of the purified antibodies were confirmed using native and reduced SDS-PAGE (refer Figure S1). Size-exclusion HPLC analysis (MAbPac™ SEC-1 column, Thermo Fischer Scientific, Waltham, MA, USA) with a flow rate of 1 mL/min was performed to verify the antibody integrity and confirm the absence of aggregates or other impurities. The concentrations of the antibodies were measured using the NanoDrop Spectrophotometer (Thermo Fisher Scientific, Waltham, MA, USA) [7].

### 2.2. In Vivo Pharmacokinetic Study

**Figure 1:**
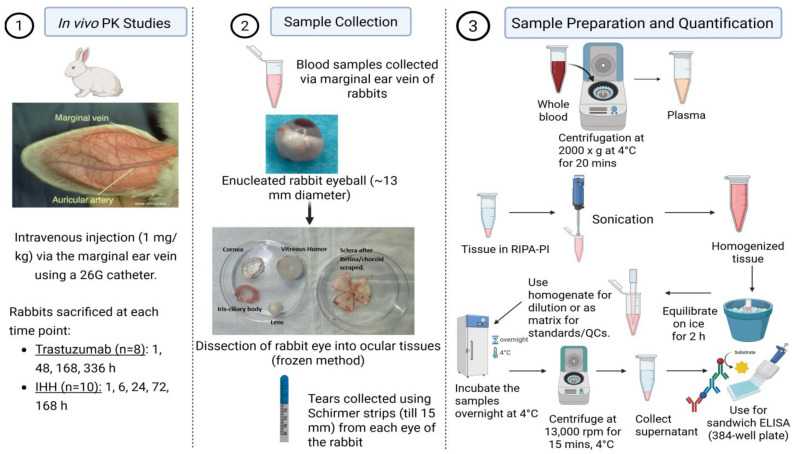
Graphical representation of experimental set-up used to assess PK of Trastuzumab and its FcRn non-binding variant (IHH) in ocular tissues and plasma in New Zealand White (NWZ) rabbits after intravenous (IV) dose of 1 mg/kg.

The in vivo PK study design and tissue preparation method has been summarized in Figure 1. The animal study was conducted in accordance with the Institutional Animal Care and Use Committee (IACUC, protocol PHC58127N). Male New Zealand White rabbits (*n* = 18; Charles River Laboratories, Wilmington, MA, USA; strain 052) that were approximately 5 months old and weighed between 2.5 and 3.0 kg were randomly divided into two study groups. Prior to dosing, animals were sedated via an intramuscular injection of acepromazine (1 mg/kg). A single intravenous bolus dose of 1 mg/kg of either wild-type trastuzumab (TS-WT) or its FcRn non-binding variant (IHH) was administered through the marginal ear vein, using a 26G catheter.

Blood and ocular tissue samples were collected at designated terminal time points following euthanasia. For TS-WT, samples were collected at 1 h, 48 h, 168 h, and 336 h post-dose, whereas for IHH, collections were made at 1 h, 6 h, 24 h, 72 h, and 168 h. At each time point, two animals were sacrificed, providing four replicate eyes (left and right from each rabbit), which allowed for the evaluation of the inter-sample and process variability associated with sample collection and preparation and immunoassay procedures [8]. No inter-eye correlation was established, the concentrations from all four eyes (two animals per time point) were averaged, and variability was expressed as the standard deviation across these measurements.

At terminal time points, approximately 1 mL of blood was collected from each animal into Eppendorf (EPP) tubes pre-loaded with 10 µL of 0.5 M EDTA to prevent coagulation. Plasma was separated by centrifugation at 2000× *g* for 20 min at 4 °C.

### 2.3. Ocular Tissue Preparation for Analysis

#### 2.3.1. Enucleation

The eyeball was enucleated following the instructions mentioned in Ahn et al. [9]. Accordingly, the eyelids were retracted by using an ocular speculum. Using a pair of pointed forceps and dissection scissors, an incision was made in the conjunctiva that is 2–3 mm posterior to the limbus. This cut was extended across the globe to loosen the globe from its ocular orbit. The cut conjunctiva was collected in an EPP tube, flash frozen using liquid nitrogen and then further stored in −80 °C until further use. Then, the extraocular muscles attached to the globes were located and cut to further dislocate the globe from the orbit. Using curved forceps, the optic nerve, which is concealed behind the globe, was located and cut using the dissection scissors. In this way, the eyeball was removed from the socket, keeping the surrounding tissues intact. If any remnants of extraocular tissues were seen to be attached to the posterior surface of the globe, they were removed using pointed forceps and sharp scissors. The globe was then rinsed with 1 mL of phosphate-buffered saline (1× PBS) to remove the surface blood, and gently blotted dry on sterile gauze.

Following enucleation, aqueous humor was collected via paracentesis using a 27-gauge needle attached to a 1 mL syringe. The needle was inserted approximately 1 mm anterior to the limbus, parallel to the iris plane, and advanced through the cornea into the anterior chamber. Up to 250 μL of aqueous humor was withdrawn per eye. The remaining enucleated globe was transferred to a 50 mL falcon tube, flash-frozen in liquid nitrogen, and stored at –80 °C until further processing [10].

#### 2.3.2. Dissection

During the dissection of the eyeball, it was separated into the vitreous humor, retina, sclera, cornea, iris–ciliary body, and lens (refer Figure S3). The dissection of the eyeball into its tissues was completed on dry ice before it was thawed. This prevented cross-contamination and easy recovery of most of the ocular tissues. While the eyeball was frozen, an incision was made at the limbus using a scalpel blade and then the cut was made across the entire globe to separate the posterior segment, comprising the sclera, retina, vitreous humor, and anterior segment, comprising the iris–ciliary body, lens, aqueous humor, and cornea. Using another pair of blade and tweezers, the retina was scraped from the underlying sclera. The iris–ciliary body and lens were separated from the cornea. All the dissected ocular tissues were collected in separate, pre-labeled tubes. The aqueous humor, vitreous humor, retina, iris–ciliary body, cornea, and conjunctiva were collected from the right and left eye of each animal. Several studies have employed a similar method for the collection of ocular tissues [8,9,11,12], demonstrating its reliability and reproducibility.

#### 2.3.3. Homogenization

The ocular tissue homogenization methods have been summarized in Table S1. For homogenization, the volume or weight of each ocular tissue was first determined by subtracting the weight of the empty EPP tube from that of the tube containing the tissue sample. Aqueous humor, due to its clear, water-like consistency, was not subjected to homogenization and was used directly for immunoassay analysis [9,12,13].

The vitreous humor samples were solubilized by adding an equal volume of phosphate-buffered saline (1× PBS) containing 1% Bovine Serum Albumin (BSA), resulting in a 2× dilution. Samples were gently rotated overnight at 4 °C, then centrifuged at 3000× *g* for 10 min to collect the supernatant for analysis [9,12,13].

Solid ocular tissues—including the retina, iris–ciliary body, lens, cornea, and conjunctiva—were homogenized using Pierce^®^ RIPA Buffer (Thermo Fisher Scientific, Waltham, MA, USA) with 1× Halt™ Protease Inhibitor Cocktail (Thermo Fisher Scientific, Waltham, MA, USA). A tissue-to-reagent ratio of 1:10 (1 g of tissue per 10 mL of buffer) was used for most tissues, while a higher dilution ratio of 1:20 was used for the cornea. The homogenization process used a Fisherbrand™ Model 120 Sonic Dismembrator (Fisher Scientific, Waltham, MA, USA) with a 1/8″ probe. Tissues underwent three pulses of sonication of 10 s on and 15 s off (ice cool down), with the sonication amplitude set to 50% (for retina) and 75% for iris–ciliary body, lens, and conjunctiva. While a less than 20 mg sample of cornea (2 mm × 2 mm) was cut and homogenized using zirconium beads (3.0 mm, Benchmark Scientific, Sayreville, NJ, USA ), homogenized using BeadBug™ microtube homogenizer (Benchmark Scientific, Sayreville, NJ, USA). Retina and iris–ciliary body homogenates were equilibrated on ice for 2 h before further dilution to 4× (total dilution = 40×) with 0.1% BSA in 1× PBS. The standards and quality controls were prepared similarly, using control ocular tissue. All the homogenates were stored at 4 °C overnight, and the supernatants were collected after centrifugation at 13,000 rpm for 15 min, 4 °C. The resulting supernatants were collected and used for immunoassays.

#### 2.3.4. Tear Extraction

**Figure 2:**
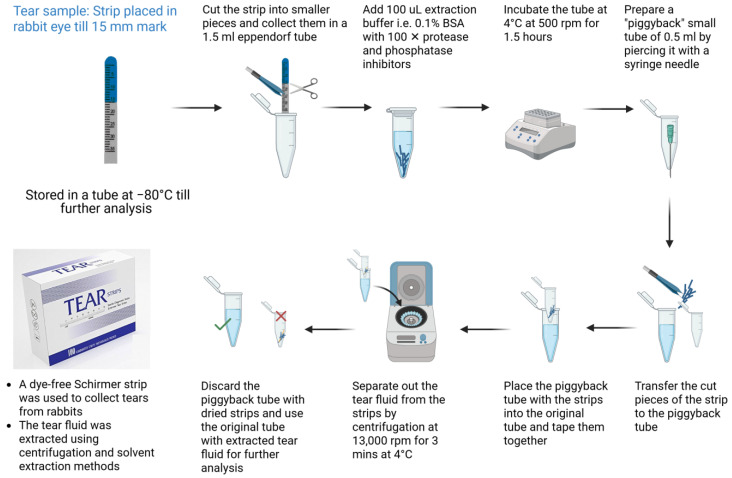
Tear extraction protocol utilizing centrifugation and solvent extraction methods.

The tear extraction procedure has been summarized in Figure 2. The Schirmer Strips (dye-free, Contacare Ophthalmics and diagnostics, Vadodara, Gujarat, India) were used for tear collection in rabbits. The strips are put in the inferior conjunctival fornix in both eyes. The tears are collected without topical anesthesia until the 15 mm mark is reached on the strip (2–3 min). After collection, the strips were placed into EPP tubes, flash-frozen in liquid nitrogen, and stored at –80 °C until further analysis. To extract the fluid from the strips, a “piggy-back” method was used, involving centrifugation and solvent-extraction methods. In this method, the strips were cut into small pieces, and 100 µL of 0.1% BSA in 1× PBS with 1× Halt™ Protease Inhibitor Cocktail (Thermo Fisher Scientific) was added to extract the tears. The samples were incubated at 1.5 h at 4 °C and 500 rpm on a shaker. Then, a 0.2 mL “piggy-back” tube with a puncture at the bottom, made using an 18-guage syringe, was prepared. After incubation, the small pieces of strips were transferred into this piggyback tube, which was added to the bigger, original EPP tube and secured with adhesive tape. The combination was centrifuged at 13,000 rpm at 4 °C for 3 min. The extracted tear fluid was collected in the larger EPP tube, while the dried strips remaining in the piggyback tube were discarded. Further, an immunoassay was performed on the test tear fluid samples [14,15].

### 2.4. Analytical Method Development

A sandwich enzyme-linked immunosorbent assay (ELISA) was employed to quantify the protein concentrations in plasma and ocular tissues, using 384-well plates (Thermo Scientific, Catalog No: 464718). For plate coating, a goat anti-human IgG (Fc-specific, cross-adsorbed) F(ab′)_2_ antibody (Bethyl Laboratories, Montgomery, TX, USA) was used as the capture antibody. It was diluted to 5 µg/mL in 20 mM Na_2_HPO_4_, and 60 µL/well of this solution was added to the plate, which was then incubated overnight at 4 °C.

The following day, the plates were brought to room temperature and washed three times each with 1× PBS containing 0.05% Tween-20 (PBS-T), followed by deionized water washes. Blocking was performed by adding 90 µL/well of 1% BSA, with incubation at room temperature for 1 h. The plates were then washed again, as described above.

Samples, standards, and quality controls (QCs) of either trastuzumab or the FcRn non-binding variant (IHH) were prepared in the relevant tissue matrices—rabbit plasma, retina, iris–ciliary body, lens, vitreous humor, and 1× PBS (used for aqueous humor and tear samples)—at concentrations ranging from 500 to 0.122 ng/mL. The QC samples were prepared independently by spiking known antibody concentrations into tissue-relevant matrices and were used to evaluate assay accuracy and precision. Each was added in triplicate (30 µL/well) to the plates and incubated at room temperature for 2 h, followed by washing.

Subsequently, 60 µL/well of goat anti-human IgG (F(ab’)_2_-specific, alkaline phosphatase-conjugated, cross-adsorbed (Bethyl Laboratories, Montgomery, TX, USA) was added as the detection antibody and diluted 1:2500 in 1× PBS-T. The plates were incubated at room temperature for 1 h before the final wash step. Detection was carried out by adding 60 µL/well of p-nitrophenyl phosphate (PNPP) substrate at a concentration of 1 mg/mL, prepared in 1× diethanolamine (DEA) buffer. Absorbance at 405 nm was measured kinetically, using a FilterMax™ F5 microplate reader (Molecular Devices, San Jose, CA, USA). Data were analyzed using a 4- or 5-parameter logistic standard curve in SoftMax^®^ Pro software (version 7; Molecular Devices, San Jose, CA, USA) [7]. All ELISA measurements, including calibration standards, quality control samples, and unknown samples, were quantified in ng/mL. For pharmacokinetic analyses and data presentation, concentrations were subsequently converted to nM using the molecular weight of IgG (150 kDa).

### 2.5. Data Analysis

Areas under the concentration–time curves (AUC0-last) for all analytes were calculated using noncompartmental analysis in MATLAB 2024b, using SimBiology 6.0 using the IV bolus route of administration for plasma PK and the extravascular route for ocular tissue PK [16]. The antibody biodistribution coefficient (%ABC) was calculated using the following formula [17]:

$$ABC=\frac{{AUC}_{Tissue}}{{AUC}_{plasma}}\times 100$$

Tissue-to-plasma (T/P) ratios were calculated at each time point by dividing the mean tissue concentration by the corresponding mean plasma concentration. These tissue-to-plasma ratios were calculated for all the tissues and plotted against time.

$$\frac{T}{P}=\frac{{Concentration}_{Tissue}}{{Concentration}_{Plasma}}$$

For statistical analysis, AUC_0–168h_ were calculated using the PKNCA package (version 0.12.1) in R with the Bailer method (sparse sampling), and the corresponding mean, standard deviation (SD), standard error (SE), and degrees of freedom (df) were obtained. Welch’s *t*-tests were performed in R on AUC values using these parameters to assess differences in absolute exposure between TS-WT and IHH.

## 3. Results

### 3.1. Antibody Production and Purification

The trastuzumab and its FcRn non-binding variant (IHH) antibody were produced by scaling up the transfected CHO-cells to a 2L flask in a shaker incubator. An approximate yield of 20 mg of antibody per liter of the cell culture media was obtained across multiple batches. In the SDS-PAGE (Figure S1), reducing and non-reducing conditions were used, which confirmed the identity and purity of the antibody. In reducing conditions, the bands corresponding to 25 kDa of F(ab)2 and 50 kDa of Fc-portion of the antibody were observed, while in non-reducing conditions, only the band corresponding to 150 kDa, the molecular weight of the antibody, was observed. Size-exclusion HPLC analysis (Figure S2) confirmed that both antibodies remained intact, with no detectable aggregation or degradation. The chromatograms showed single dominant peaks for each antibody (retention times ~11.9 min for both TS-WT and IHH), accounting for over 99% of the total peak area, indicating high purity and structural integrity.

### 3.2. Analytical Method Development

Figures S4 and S5 show typical ELISA standard curves and quality controls (QCs) for trastuzumab and IHH in the ocular tissues, including the retina, iris–ciliary body, vitreous humor and lens. The lower limit of quantitation was 0.122–0.488 ng/mL for all the tissues and the standard curves had R2 of ≥0.95 in the SoftMax^®^ Pro software, using a 4/5-parameter equation. A standard curve prepared in 1× PBS was employed for the analysis of tear and aqueous humor samples, due to the limited availability of the corresponding blank control tissues. Similarly, a lens-derived standard curve was used as a surrogate matrix for cornea and conjunctiva analyses, owing to sample scarcity and homogenization constraints. However, blank control samples of cornea (*n* = 5), conjunctiva (*n* = 5), and aqueous humor (*n* = 5) exhibited no detectable signal, confirming the absence of background interference.

### 3.3. Tear Extraction

To prevent potential interference from dyes in the analysis of the drug, dye-free Schirmer strips were used for tear collection. Tear extraction was performed using 100 µL of 0.1% BSA in 1× PBS supplemented with 1× Halt™ Protease Inhibitor Cocktail to prevent protein degradation. To evaluate the recovery efficiency of this method, 12 blank Schirmer strips were spiked with 10 µL of trastuzumab at a known concentration of 20 ng/mL and analyzed. Following a 10× dilution, the average recovered concentration of trastuzumab was 17.25 ng/mL (recovery rate = 86.25%).

To further confirm recovery, another antibody, mouse IgG2a, was tested at two concentrations (100 ng/mL and 50 ng/mL). A volume of 5 µL of each solution was used to spike 12 blank strips (*n* = 6 per concentration). The average recovered concentrations were 62.13 ng/mL (recovery rate = 62.13%) and 36.49 ng/mL (recovery rate = 72.98%) for the 100 ng/mL and 50 ng/mL groups, respectively. The lower recovery rate for mouse IgG2a may be attributed to the reduced spike volume, highlighting the importance of a sufficient tear volume for accurate quantification.

A 10× dilution factor was determined based on the average tear volume absorbed by the strip, which was calculated by weighing blank strips before and after tear collection. This was necessary because, in our protocol, tears were collected to a fixed length (15 mm) on the Schirmer strip, unlike in clinical practice, where collection is typically time-based (e.g., 30 or 60 s). The average weight gain of 16 tear strips was approximately 12 mg, corresponding to an estimated tear volume of 12 µL. This result agrees with an in vitro wetting experiment reported in the literature, enabling the conversion of in vivo tear strip wetting length into volumes of tear production [18]. Furthermore, blank tear strips (*n* = 6) showed no detectable signal, confirming the absence of background interference in the assay.

### 3.4. Comparative Pharmacokinetics of Trastuzumab and FcRn Non-Binding Variant (IHH) in Plasma and Ocular Tissues

**Figure 3:**
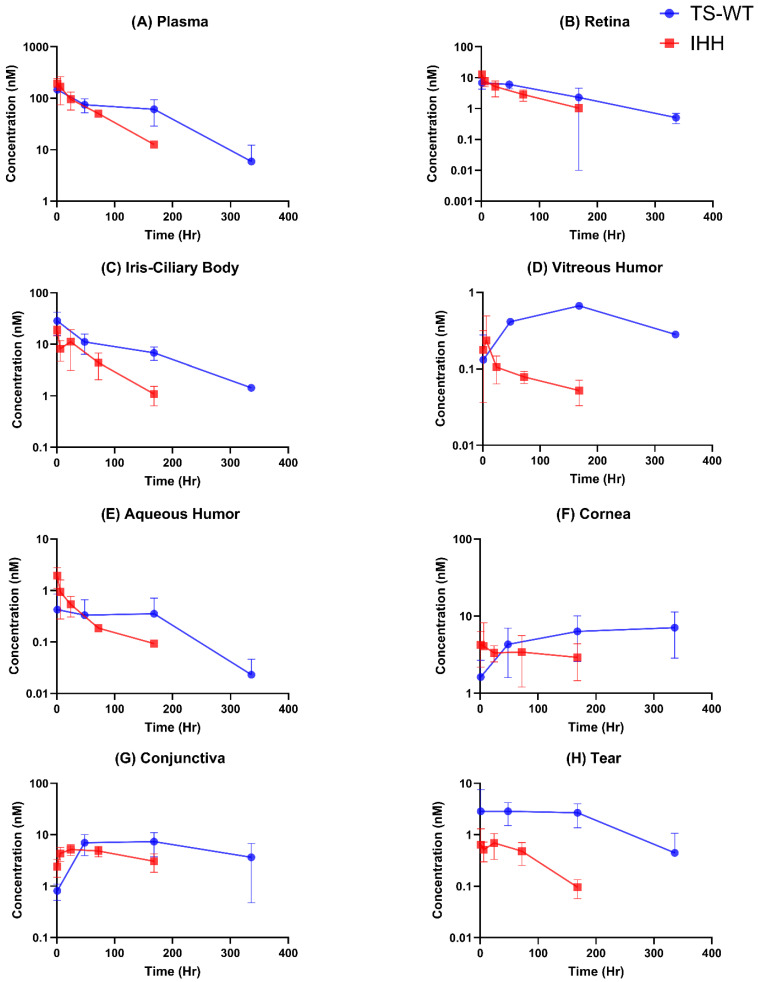
Trastuzumab (blue) and FcRn-non-binding trastuzumab (IHH, red) PK profiles. Each data point represents the mean ± S.D.

Figure 3 depicts the pharmacokinetic profiles of wild-type trastuzumab (TS-WT) and its FcRn non-binding variant (IHH) in plasma and seven distinct ocular tissues over a period of 336 h (14 days) for TS-WT and 168 h (7 days) for FcRn non-binding trastuzumab (IHH), following intravenous administration in New Zealand White rabbits at a 1 mg/kg dose.

#### 3.4.1. Systemic Exposure

Figure 3A shows that while TS-WT and IHH start with similar initial concentrations, their elimination profiles differ significantly. Individual animal plasma concentration–time profiles for TS-WT are shown in Supplementary Figure S7. TS-WT maintains a higher concentration throughout the study period, whereas IHH shows faster clearance, which is consistent with impaired FcRn binding, leading to a reduced systemic half-life. Notably, TS-WT shows an unexpected 10-fold drop in plasma concentrations from 70 nM at 168 h to 6 nM at 336 h. This abrupt decline in concentrations is inconsistent with typical antibody elimination kinetics, as well as tissue PK profiles, where trastuzumab generally maintains better retention through 336 h. The observed drop also suggests a much shorter half-life, whereas therapeutic antibodies like trastuzumab typically exhibit bi-exponential elimination with a terminal half-life of approximately 20–25 days, due to FcRn recycling.

#### 3.4.2. Intraocular Distribution

The retina PK profile (Figure 3B) closely parallels the plasma profile, with both antibodies showing similar initial concentrations and TS-WT maintaining higher levels over time. However, the large error bars at the 168 h time point for TS-WT indicate substantial variability in retinal concentrations. As with the retina, the iris–ciliary body (Figure 3C) elimination profiles closely reflect plasma trends, with IHH clearing more rapidly (1 nM at 168 h) compared to TS-WT (7 nM at 168 h). Notably, the ICB also exhibited the highest initial antibody concentrations among all the tissues analyzed.

In the vitreous humor (Figure 3D), both antibodies begin at similar, relatively low concentrations compared to other tissues. Over time, TS-WT shows a marked increase in concentration, in contrast to the progressive decline observed with IHH. Unlike its plasma profile, TS-WT accumulates significantly in the vitreous, reaching levels at 168 h that are ~3-fold higher than the initial concentrations and ~10-fold higher than IHH—representing the most pronounced difference between the two antibodies across all tissues examined. In the aqueous humor (Figure 3E), IHH initially shows higher concentrations, but TS-WT maintains higher concentrations at later time points. As with the vitreous humor, the overall concentrations in the aqueous humor are among the lowest across the ocular tissues examined.

In the cornea (Figure 3F), TS-WT shows progressive accumulation over time, while IHH maintains stable concentrations over 168 h. In the conjunctiva (Figure 3G), like the cornea, both antibodies show accumulation over time. While IHH peaks earlier and at lower concentrations, TS-WT sustains higher concentrations throughout later time points. In tears (Figure 3H), TS-WT consistently shows higher concentrations, with a 10-fold difference from IHH by 168 h.

Thus, across most tissues, the wild-type trastuzumab (TS-WT) demonstrates higher concentrations and sustained presence compared to the FcRn non-binding variant (IHH), highlighting the critical role of FcRn binding in maintaining antibody levels both systemically and within the ocular tissue. The concentrations in the lens were below the lower limit of quantification (LLOQ) and therefore not detectable; consequently, the pharmacokinetic (PK) profile for the lens was not reported.

### 3.5. Ocular Tissue-to-Plasma Concentration Ratios

**Figure 4:**
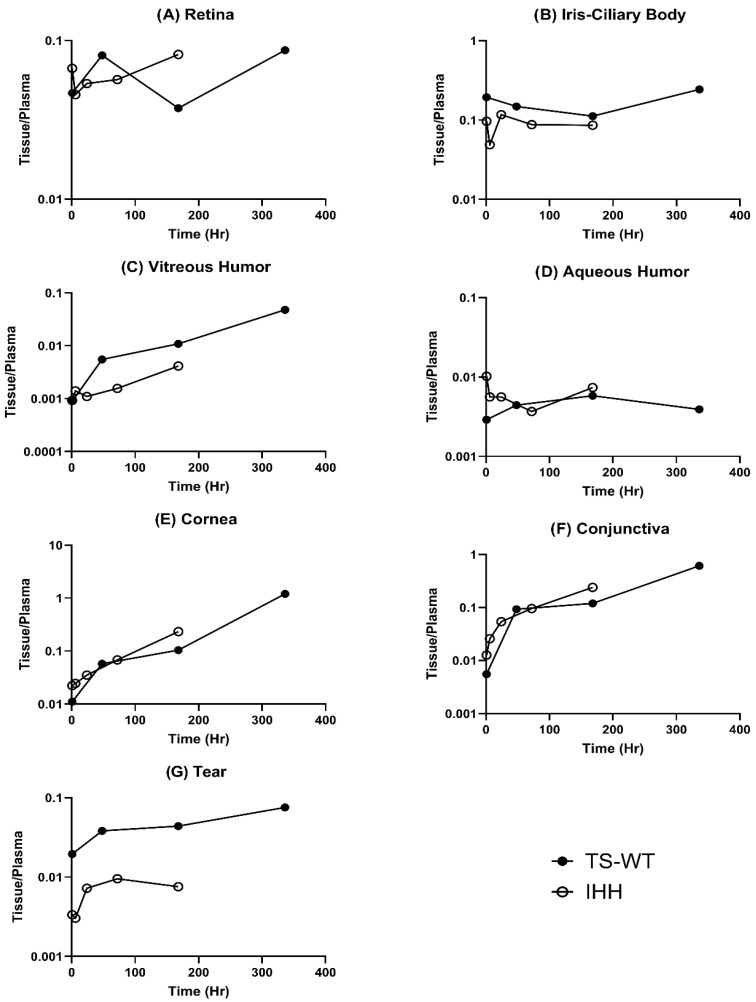
Tissue over plasma concentration ratios of trastuzumab and FcRn non-binding Trastuzumab (IHH).

To normalize differences in systemic exposure and better understand the tissue distribution patterns, we analyzed the tissue-to-plasma concentration ratios for wild-type trastuzumab (TS-WT) and its FcRn non-binding variant (IHH) across various ocular tissues (Figure 4).

Both antibodies, TS-WT and IHH, showed relatively similar tissue-to-plasma (T/P) ratios in the retina (Figure 4A) and demonstrated the highest initial T/P ratios in the iris–ciliary body (Figure 4B) among all ocular tissues. In the vitreous humor (Figure 4C), TS-WT exhibited an approximately 3-fold higher tissue-to-plasma (T/P) ratio than IHH by 168 h, while aqueous humor (Figure 4D) exhibited minimal differences between the antibodies. In the cornea and conjunctiva (Figure 4E and Figure 4F, respectively), TS-WT and IHH exhibited comparable tissue-to-plasma (T/P) ratios up to 168 h, with similar time-dependent increases and no consistent separation between the two antibodies during this period. In tear fluid (Figure 4G), TS-WT exhibited consistently higher tissue-to-plasma (T/P) ratios than IHH up to 168 h, with approximately 4–6-fold higher values observed across the sampled time points. These patterns suggest that FcRn binding does not produce a uniform increase in trastuzumab retention across ocular tissues. Instead, the relative retention was comparable between TS-WT and IHH in the cornea and conjunctiva, modestly higher in the vitreous humor despite low absolute exposure, and most clearly increased in tear fluid and the iris–ciliary body, indicating tissue-specific FcRn-associated effects, rather than a global effect across the eye.

### 3.6. Antibody Biodistribution Coefficients (ABC)

Table 1 presents the area under the concentration–time curve (AUC_0-last_) and antibody biodistribution coefficient (ABC) values, which quantitatively represent the overall drug exposure and relative tissue distribution, respectively. To enable equivalent FcRn-related comparisons between TS-WT and IHH, AUC_0–168h_ and the corresponding ABC_0–168h_ values were used as the primary analysis window. Using this matched window, TS-WT achieved approximately 2-fold higher systemic exposure than IHH, which reflects the expected impact of FcRn binding on prolonging the systemic circulation of antibodies. In ocular tissues, TS-WT consistently achieved a higher absolute exposure than IHH. The highest absolute exposure for trastuzumab was observed in the iris–ciliary body, followed by the conjunctiva, cornea and retina. The vitreous humor, tear fluid and aqueous humor exhibited substantially lower absolute exposure. For IHH, the rank order of absolute tissue exposure paralleled that of TS-WT.

The antibody biodistribution coefficient (ABC), calculated as the ratio of tissue AUC to plasma AUC (expressed as a percentage), revealed striking differences in the relative distribution patterns between the two antibodies. A comparison of TS-WT ABC values calculated using AUC_0–168h_ versus AUC_0–336h_ indicated that the majority of ocular tissues exhibited a similar relative distribution across the two analysis windows, suggesting that late-time plasma variability had a limited impact on the overall tissue biodistribution estimates (Table 1). While the retinal distribution remained comparable between antibodies (ABC_0–168h_: 5.96% vs. 5.51%), TS-WT demonstrated increased relative distribution to the vitreous humor and tear fluid (3.5- and 5.5-fold higher ABC values, respectively) compared to IHH. The iris–ciliary body showed the highest relative distribution for both antibodies, with TS-WT exhibiting higher accumulation (14.95% vs. 8.89%). The ABC_0–168h_ values for the cornea were similar between TS-WT and IHH (5.76% vs. 5.57%), as were the ABC_0–168h_ values for the conjunctiva (7.71% vs. 7.21%), indicating a comparable relative distribution of the two antibodies in these tissues. Aqueous humor was uniquely characterized by slightly higher relative IHH distribution. These findings indicate that FcRn enhances antibody accumulation in ocular tissues, such as the iris–ciliary body, and tears, with minimal effects on the retina, cornea, conjunctiva, and aqueous humor distribution. Vitreous humor exhibited low absolute exposure and only a modest dependence on FcRn, suggesting that the observed differences are influenced primarily by the limited access of the systemically administered antibodies, rather than direct FcRn-mediated transport.

### 3.7. Statistical Hypothesis Testing

**Figure 5:**
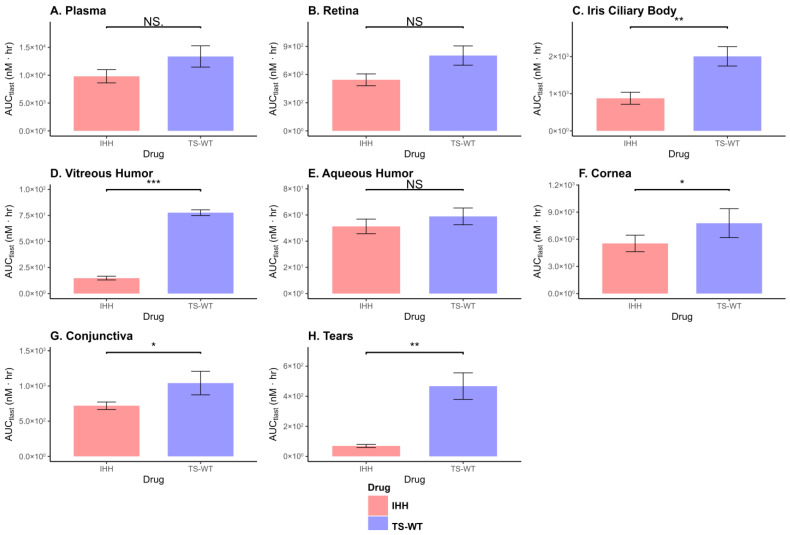
Comparison of AUC_0–168h_ for IHH and TS-WT antibodies across plasma and ocular tissues following IV administration of a 1 mg/kg dose in New Zealand White rabbits. Bars represent mean AUC_0–168h_ values, and error bars indicate standard deviation (SD). Statistical significance between IHH and TS-WT within each tissue was assessed using Welch’s *t*-test. *p*  <  0.05 (*), *p * <  0.01 (**), *p*  <  0.001 (***), and *p*  <  0.0001 (****); NS, not significant.

Statistical comparisons (Figure 5) based on absolute exposure (AUC_0–168h_) revealed no significant differences between TS-WT and IHH in the plasma or retina. In contrast, TS-WT demonstrated significantly higher AUCs in the iris–ciliary body and vitreous humor, indicating increased absolute tissue exposure, relative to the FcRn non-binding variant. Significantly higher AUCs for TS-WT were also observed in the cornea, conjunctiva, and tear fluid, which was consistent with the enhanced ocular exposure in these tissues. These statistical comparisons were performed using Welch’s *t*-tests on AUC values derived from sparse sampling, with *n* = 2 animals per time point. Therefore, with richer plasma and tissue sampling, additional differences, particularly at later time points, may become apparent. Importantly, while AUC-based analyses indicate differences in absolute exposure, the ABC values were comparable between TS-WT and IHH for ocular tissues such as the cornea and conjunctiva, suggesting that increased tissue exposure for TS-WT largely reflects proportional increases relative to systemic plasma exposure, rather than changes in relative tissue distribution.

## 4. Discussion

The current study reports a PK study of wild type trastuzumab and its FcRn non-binding variant (IHH) in New Zealand White rabbits after an IV bolus dose of 1 mg/kg. The concentrations in plasma and ocular tissues like the retina, iris–ciliary body, vitreous humor, aqueous humor, cornea, conjunctiva, and tears were detected using sandwich ELISA.

While the ocular PK of biologics in compartments such as the aqueous humor, vitreous humor, and retina has been extensively studied following intravitreal (IVT) administration [11,12,13,19], their ocular disposition after systemic administration remains less well-characterized [8,20]. Furthermore, most existing studies have been limited to a subset of ocular tissues, often omitting regions such as the lens, cornea, conjunctiva, and iris–ciliary body. In contrast, our study presents a comprehensive PK assessment across a broader range of ocular tissues, including these regions as well as tears, providing a more holistic view of antibody distribution following systemic exposure. Tear sampling for PK analysis has previously employed methods such as Schirmer strips and capillary tubes, primarily in the context of topically administered small-molecule drugs like ofloxacin [21] and dexamethasone [22]. However, to our knowledge, this is the first study to report monoclonal antibody (mAb) concentrations in tears following systemic administration.

While numerous studies have explored the ocular PK of lower molecular weight antibody fragments (e.g., Fab, F(ab′)_2_, scFv) [11,13,19,20], due to their enhanced tissue penetration, the role of the neonatal Fc receptor (FcRn) in the biodistribution of full-length antibodies to ocular tissues has not been fully elucidated. Gadkar et al. [11] investigated the ocular PK of a non-targeting anti-gD IgG and its FcRn non-binding variant in rabbits following intravitreal (IVT) administration, reporting concentrations in ocular tissues such as the retina, aqueous humor, and vitreous humor. Although they also included plasma PK profiles following IV dosing, the ocular tissue concentrations were not assessed under these conditions. Notably, our dose-normalized plasma PK profiles closely align with those reported by Gadkar et al., except for a divergence at the 336 h time point for the wild-type antibody (Figure S6). This phenomenon could potentially be attributed to the development of anti-drug antibodies (ADAs) against the humanized trastuzumab in rabbits, accelerating its clearance at later time points. Future studies should incorporate ADA measurements to address this possibility [23]. 

As expected, the FcRn non-binding variant (IHH) exhibited substantially accelerated systemic clearance compared to wild type trastuzumab, which was consistent with the established role of FcRn in extending the circulatory half-life of IgG antibodies. Our findings reveal distinct tissue-specific patterns in how FcRn influences antibody distribution and retention across various ocular compartments. The iris–ciliary body demonstrated the highest absolute exposure (AUC) and relative distribution (ABC) for both antibodies, which is consistent with its high vascularity and reported high FcRn expression on the non-pigmented epithelium layer and vascular endothelium of the iris and ciliary body [3,24]. The significant difference in ABC values between TS-WT and IHH (14.18% vs. 8.89%) suggests that FcRn actively contributes to antibody accumulation in this tissue beyond what would be expected from systemic exposure differences alone.

Interestingly, the retinal distribution showed minimal dependence on FcRn binding, with nearly identical ABC values for both antibodies (5.44% vs. 5.51%). The layers of retina include (from outer to inner): the retinal pigment epithelium (RPE), photoreceptor layer, external limiting membrane (junction between muller cells and photoreceptors), outer nuclear layer, outer plexiform layer, inner nuclear layer, inner plexiform layer, ganglion cell layer, nerve fiber layer, and inner limiting membrane [25]. While the choroidal vasculature supplies the outer retina, including RPE and photoreceptors, the inner retinal vasculature supplies the inner nuclear to the nerve fiber layers of the retina. Thus, the outer blood–retinal barrier (oBRB) comprises tight junctions of RPE cells, with the basal side facing the choriocapillaris and Bruch’s membrane and the apical side facing the photoreceptors. The inner blood–retinal barrier (iBRB) is formed primarily by inner retinal vasculature, i.e., tight junctions of retinal capillary endothelial cells. FcRn is expressed in the retinal capillary endothelium, as well as choroidal vasculature [26]. However, the expression of FcRn on RPE and other cell layers in the retina is debatable, with human and porcine RPE cells being detected as being positive for FcRn expression, using mRNA analysis, PCR techniques, Western blot and immunohistochemistry [27,28], while some studies in rats did not detect FcRn in the RPE-choroid complex [24]. This discrepancy might be due to species differences or methodological variations. However, BRB significantly restricts the access of systemically administered drugs, including IgG antibodies, to the eye [29]. Further, BRB shares similarities with the blood–brain barrier, with studies examining the influence of FcRn on IgG distribution to the brain having found no significant differences in brain-to-plasma concentration ratios between the control and FcRn-knockout mice [30]. Thus, FcRn plays an important role in eliminating intravitreally administered full-length IgGs across the blood–retinal barrier into the systemic blood system [29]. Therefore, rather than facilitating systemic IgG entry, FcRn appears to have a recycling function in the retina [27]. This suggests that the retinal penetration of systemically administered IgGs is likely governed predominantly by passive diffusion or FcRn-independent transport mechanisms, leading to nearly identical ABC values between both the antibodies.

Using the matched AUC_0–168h_ window, the relative distribution of TS-WT to the cornea and conjunctiva was comparable to that of IHH, with similar ABC values observed for the cornea (5.76% vs. 5.57%) and conjunctiva (7.71% vs. 7.21%). These findings suggest that, within the primary analysis window, FcRn binding does not result in a pronounced difference in the relative antibody distribution to these tissues. Nevertheless, FcRn expression has been observed on corneal epithelial and endothelial cells, where FcRn is co-localized with Fcγ receptors [9,17]. This co-expression may suggest coordinated functions in antibody trafficking across the corneal barriers. Similarly, FcRn expression in conjunctiva has been reported for the conjunctival epithelium and lymphatic vessels. Notably, conjunctival endothelial cells lack blood–retinal barrier-like tight junctions, and hence FcRn expressed in conjunctival lymphatic vessels is hypothesized to mediate the efflux of IgG or antigen-IgG complexes from the conjunctival space [9]. Aqueous humor showed minimal FcRn dependence and overall low absolute exposure, which was consistent with the restricted accessibility of this compartment, due to the blood–aqueous barrier (formed by tight junctions between non-pigmented ciliary epithelial cells and iris endothelial cells) and aqueous humor drainage through trabecular meshwork and uveoscleral outflow, leading to the rapid clearance of antibodies from this chamber, irrespective of FcRn binding [31]. However, it was observed that at the earliest time points, the concentrations observed for the IHH in the vitreous humor, aqueous humor, cornea, and conjunctiva were higher than TS-WT. All these tissues belong to the anterior segment of the eye. Since aqueous humor demonstrated the earliest and highest difference between the two antibodies, it is possible that the observed increase in antibody exposure in the other three tissues may stem from changes in this compartment. As such, we hypothesize that FcRn may play some role in the secretion of antibodies in the aqueous humor, where binding to FcRn may help to recycle the antibodies from the ciliary processes to prevent their loss, and lack of FcRn binding may result in higher ultrafiltration/secretion of antibodies from ciliary processes to aqueous humor. However, such a hypothesis needs to be further validated.

The 5.5-fold higher ABC value in tears is especially notable, as it suggests that FcRn may facilitate antibody secretion into the tear fluid. Endogenous tear p6roteins can be classified into 2 main categories: proteins produced by the lacrimal glands (including lysozyme, lactoferrin, and IgA) and a small proportion of serum proteins derived from conjunctival capillaries, including albumin, transferrin, IgG, and IgM. While secretory IgA is the primary immunoglobulin in tears and is actively secreted by the myoepithelial cells of the lacrimal gland, IgG is present at much lower concentrations [32]. In humans, endogenous IgG concentrations in tears are relatively low: approximately 10 µg/mL in healthy individuals, as reported by Donshik et al. [33]. In contrast, the plasma IgG levels are around 10 mg/mL [34], resulting in an approximate tear-to-plasma IgG ratio of 1:1000.

Thus, systemically administered IgG can reach the tear fluid via conjunctival blood vessels and epithelium. Leakage through the blood–tear barrier (comprising tight junctions of the corneal and conjunctival epithelium) allows for passive diffusion of high-molecular-weight proteins like IgG (~150 kDa), especially under inflammation or elevated systemic levels [35]. FcRn expression in ocular tissues, especially the conjunctiva [24], can facilitate the active transport of IgG and mAbs into the tear film following systemic administration. This enhances their levels in tear fluid beyond what would be expected from passive diffusion alone. The neonatal Fc receptor (FcRn) has been detected in both rodents and human lacrimal gland epithelial cells [36]. FcRn is known to mediate the bidirectional transport of IgG across epithelial barriers (e.g., in the lung, gut, and placenta) [37], suggesting a similar function could occur in the lacrimal gland. Studies in FcRn knockout mice show reduced IgG levels in ocular secretions, including tear fluid, supporting that FcRn actively transports IgG from blood into tears [38]. This suggests that the lacrimal gland is not just a passive fluid producer, but may actively secrete IgG into the tear film.

Our findings have several important implications for the development of antibody-based therapeutics for ocular diseases. First, a majority of antibody–drug conjugates (ADCs), regardless of their target or payload, exhibit ocular toxicity, particularly in the corneal epithelium, including corneal keratopathy. However, the mechanism by which ADCs enter the corneal epithelium remains poorly understood. While non-specific macropinocytosis is believed to account for much of this toxicity, the exact route of ADC entry into the cornea is not fully elucidated. It can be hypothesized that ADCs may access the cornea via exposure of the corneal surface to tears, vascularized regions of the limbus, or through FcRn-mediated transport in corneal epithelial cells [39]. Our observed tear concentrations and FcRn-dependent distribution patterns in ocular tissues such as the iris–ciliary body, cornea, and conjunctiva support further investigation into these mechanisms. Second, the measured concentrations of TS-WT and IHH across various ocular tissues provide a valuable foundation for developing a physiologically based pharmacokinetic (PBPK) model that incorporates a detailed mechanistic ocular compartment [40]. This model would enhance our understanding of tear turnover dynamics, FcRn expression within the eye, and the transport of monoclonal antibodies across multiple blood–ocular barriers. Third, in ocular diseases such as choroidal neovascularization (CNV), FcRn expression is upregulated, particularly in the retina, leading to the accelerated clearance of intravitreally administered biologics like bevacizumab. This suggests that the design of FcRn non-binding mAbs may be a promising strategy to reduce rapid elimination and improve therapeutic efficacy in CNV patients [29].

However, this study has several limitations that present opportunities for future research. First, in accordance with the 3Rs principle (Replacement, Reduction, Refinement) for ethical animal use in preclinical studies, only two animals were sacrificed per time point, yielding four ocular samples at each time point. While this terminal sampling approach provided comprehensive tissue distribution data, serial sampling from the same animal, such as through aqueous humor tapping and multiple plasma collections, could offer more detailed PK information while reducing inter-animal variability. Second, although our tissue collection method was designed to minimize cross-contamination between ocular compartments, the observed inter-animal variability, particularly in certain tissues and at specific time points, highlights the inherent challenges in ocular pharmacokinetic studies. This variability may be attributed to individual differences in vascular permeability, tissue perfusion, and potentially in FcRn expression levels. Additionally, whole-body perfusion, including perfusion via the ophthalmic artery, was not performed prior to ocular tissue collection. Perfusion in rabbits is technically challenging, requires substantial expertise, and may disrupt the fragile vascular and epithelial structures of the ocular tissues. This approach is consistent with common practice in previously published rabbit ocular pharmacokinetic studies [8,11,12]. As a result, some contributions from residual blood or plasma cannot be excluded, particularly in highly vascularized tissues such as the iris–ciliary body. While this may influence absolute concentration estimates, comparative analyses between TS-WT and IHH within the same tissue are expected to be minimally affected, as both antibodies underwent identical collection and processing procedures. Third, while the tear fluid was collected using Schirmer strips, this method has several limitations. These include the stimulation of reflex tears during collection, which can dilute antibody concentrations, and the adsorption of mAbs onto the cellulose material of the strips, resulting in low recovery and underestimation of the true concentrations [15]. Finally, anti-drug antibodies (ADAs) were not quantified in this study. The use of rabbits as a non-rodent preclinical model for biologics has recently been questioned due to the frequent development of ADAs and the occurrence of severe ocular inflammation in this species, confounding the results [23]. Future studies should consider the impact of immunogenicity and explore strategies to mitigate ADA-related complications when evaluating biologics in ocular models.

## 5. Conclusions

This study provides a comprehensive pharmacokinetic evaluation of wild-type trastuzumab and its FcRn non-binding variant (IHH) across systemic and ocular tissues in New Zealand White rabbits following intravenous administration. By quantifying the antibody concentrations in a wide range of ocular tissues—including the cornea, conjunctiva, iris–ciliary body, retina, and tear fluid—our findings enhance the current understanding of FcRn-mediated distribution mechanisms within the eye. The higher ABC values observed for TS-WT compared to IHH in the iris–ciliary body and tear fluid indicate tissue-specific FcRn-associated effects on ocular antibody distribution, whereas ABC values were comparable between the antibodies in the cornea, conjunctiva, retina, and aqueous humor. The novel detection of systemically administered monoclonal antibodies in tears opens new avenues for exploring tear fluid as a non-invasive matrix for monitoring ocular drug exposure. These results have important implications for optimizing antibody-based therapies for ocular diseases, guiding the safety assessments of antibody–drug conjugates (ADCs) that exhibit corneal toxicity and informing physiologically based pharmacokinetic (PBPK) model development. Nonetheless, limitations such as inter-animal variability, potential immunogenicity, and challenges with tear sampling highlight the need for refined methodologies and further investigation. Future studies incorporating ADA assessments, alternative animal models, and improved sampling strategies will be essential to validate and expand upon these findings.

## Figures and Tables

**Table 1:** Area under the curve (AUC, nM × h) and antibody biodistribution coefficient (ABC,%) of trastuzumab and its FcRn non-binding variant (IHH).

	TRASTUZUMAB (TS-WT)				FcRn Non-Binding (IHH)	
Tissue	AUC_0–336h_ (nM × h)	ABC_0–336h_ (%)	AUC_0–168h_ (nM × h)	ABC_0–168h_ (%)	AUC_0–168h_ (nM × h/L)	ABC_0–168h_ (%)
Plasma	1.91 × 10^4^		1.35 × 10^4^		9.99 × 10^3^	
Retina	1.04 × 10^3^	5.44	8.06 × 10^2^	5.96	5.50 × 10^2^	5.51
Iris–Ciliary Body	2.71 × 10^3^	14.18	2.02 × 10^3^	14.95	8.88 × 10^2^	8.89
Vitreous Humor	1.57 × 10^2^	0.82	7.77 × 10^1^	0.58	1.49 × 10^1^	0.15
Aqueous Humor	9.09 × 10^1^	0.47	5.91 × 10^1^	0.44	5.23 × 10^1^	0.52
Cornea	1.91 × 10^3^	9.96	7.79 × 10^2^	5.76	5.56 × 10^2^	5.57
Conjunctiva	1.96 × 10^3^	10.26	1.04 × 10^3^	7.71	7.21 × 10^2^	7.21
Tear	7.31 × 10^2^	3.82	4.69 × 10^2^	3.47	6.98 × 10^1^	0.70

## Data Availability

The datasets generated in the current study are available from the corresponding author upon reasonable request.

## Supplementary Materials

The following supporting information can be downloaded at: https://www.mdpi.com/article/10.3390/antib15020027/s1, Figure S1: SDS-PAGE analysis of trastuzumab and IHH; Figure S2: Size-exclusion chromatography of Trastuzumab and IHH; Figure S3: Dissection of rabbit eyeball into ocular tissues; Figure S4: Representative ELISA standard curves for trastuzumab; Figure S5: Representative ELISA standard curves for IHH; Figure S6: Dose-normalized and superimposed plasma PK profiles of trastuzumab and IHH with literature reported; Figure S7: Individual-animal plasma concentration–time profiles of wild-type trastuzumab (TS-WT); Table S1: Ocular tissue homogenization methods.
