# Supplementary material for: The Effect of FcRn Binding on Ocular Disposition of Monoclonal Antibodies
Source: Antibodies (Basel). 2026 Mar 25;15(2):27. doi: 10.3390/antib15020027 (PMC13113779; doi:10.3390/antib15020027)
Supplement: Supplementary file 1 [file antibodies-15-00027-s001.zip › antibodies-4094370-supplementary.pdf]

Supplementary Material

The Effect of FcRn on Ocular Disposition of Monoclonal Antibodies

Sanika Naware, Saurav Kulkarni, Sahil Salvi , Dhvani Patel and Dhaval K Shah*

Department of Pharmaceutical Sciences, School of Pharmacy and Pharmaceutical Sciences, The State University of New York at Buffalo, Buffalo, NY 14214-8033, USA; snaware@buffalo.edu (S.N.); spkulkar@buffalo.edu (S.K.); sahilsal@buffalo.edu (S.S.); dhvanipr@buffalo.edu (D.P.)

* Correspondence: (D.K.S) dshah4@buffalo.edu ; Tel.: +1-716-645-4819

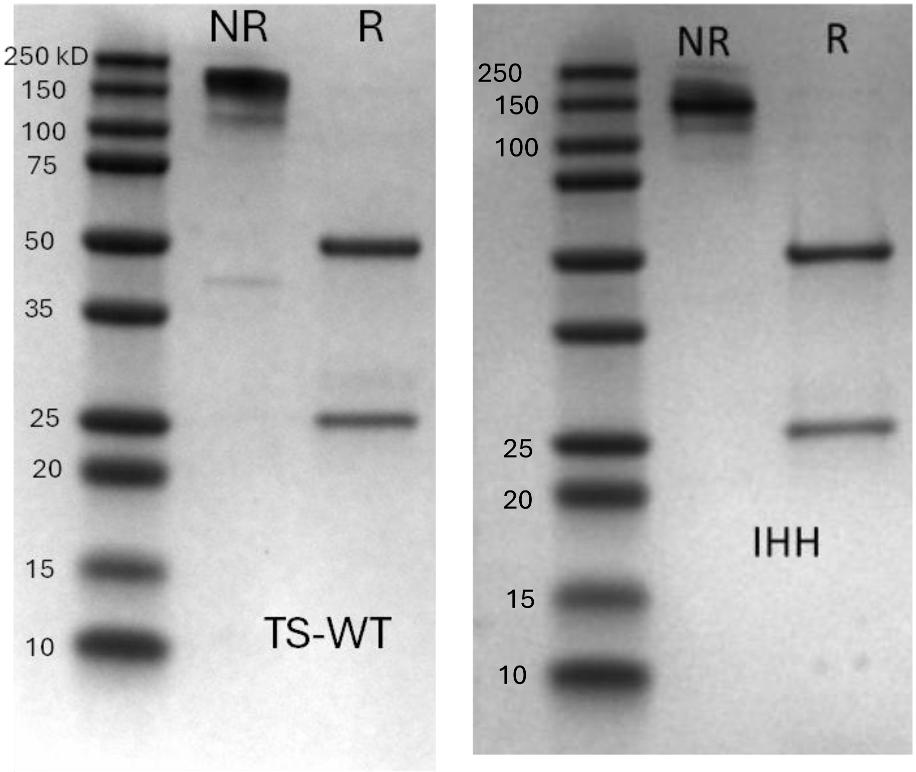

Supplementary Figure S1: SDS-PAGE analysis of trastuzumab and IHH.

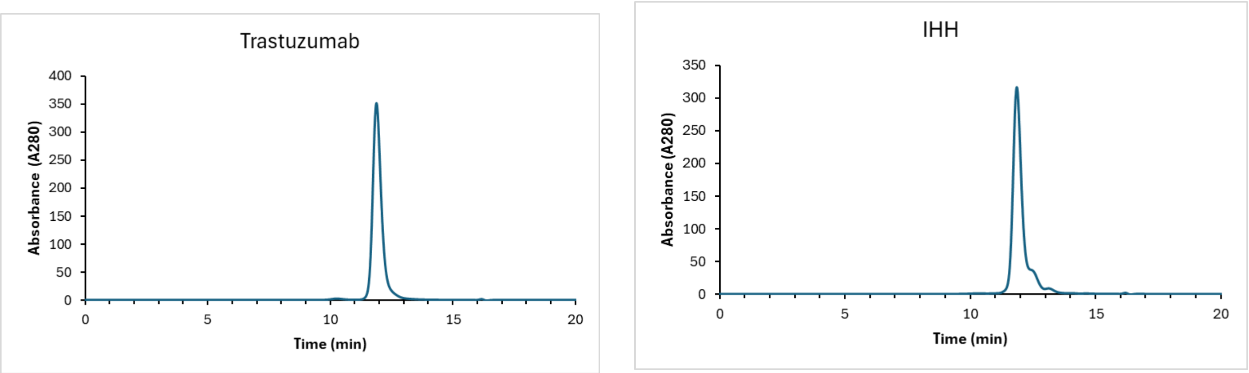

Signal 2: DAD1 E, Sig=280,16 Ref=360,100

Peak #	RetTime [min]	Type	Width [min]	Area [mAU*s]	Height [mAU]	Area %
1	10.267	BB	0.4538	59.28777	1.95481	0.6657
2	11.890	BB	0.3770	8819.73437	351.12863	99.0346
3	16.178	BV	0.2076	26.68942	2.08203	0.2997
Totals :				8905.71156	355.16547	

Signal 2: DAD1 E, Sig=280,16 Ref=360,100

Peak #	RetTime [min]	Type	Width [min]	Area [mAU*s]	Height [mAU]	Area %
1	11.862	BB	0.3975	8476.84863	315.50137	97.7295
2	13.130	BB	0.3428	169.59958	7.36181	1.9553
3	16.193	BV	0.2043	27.34146	2.15136	0.3152
Totals :				8673.78967	325.01454	

Supplementary Figure S2: Size exclusion chromatography of trastuzumab and IHH.

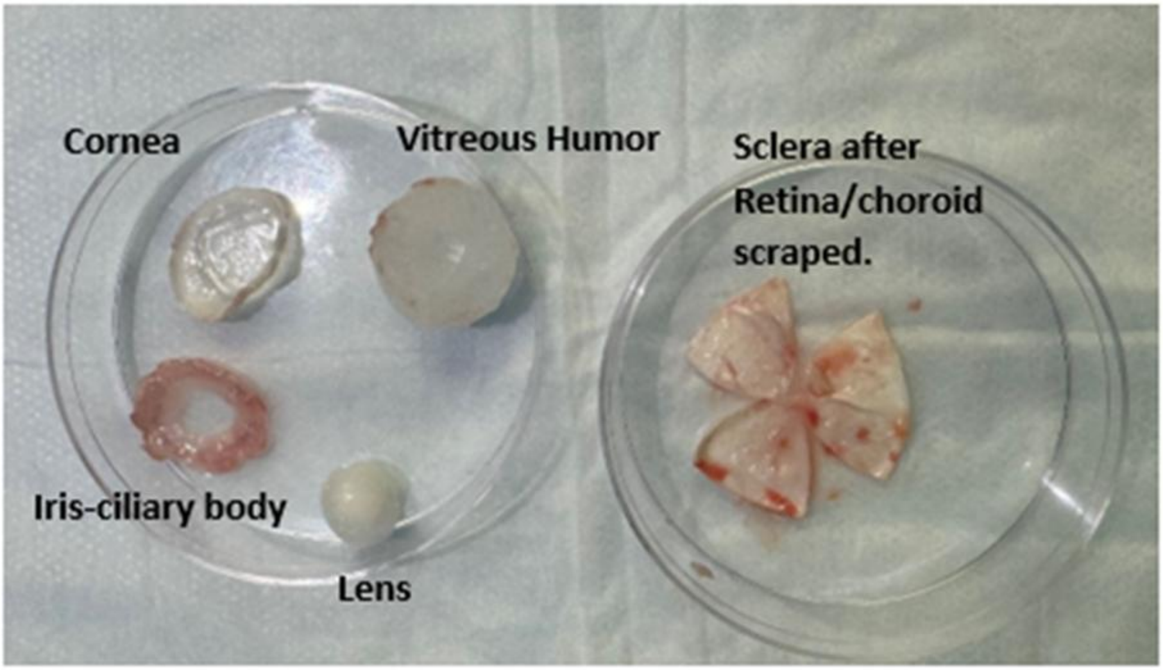

Supplementary Figure S3: Dissection of rabbit eyeball into ocular tissues.

Trastuzumab

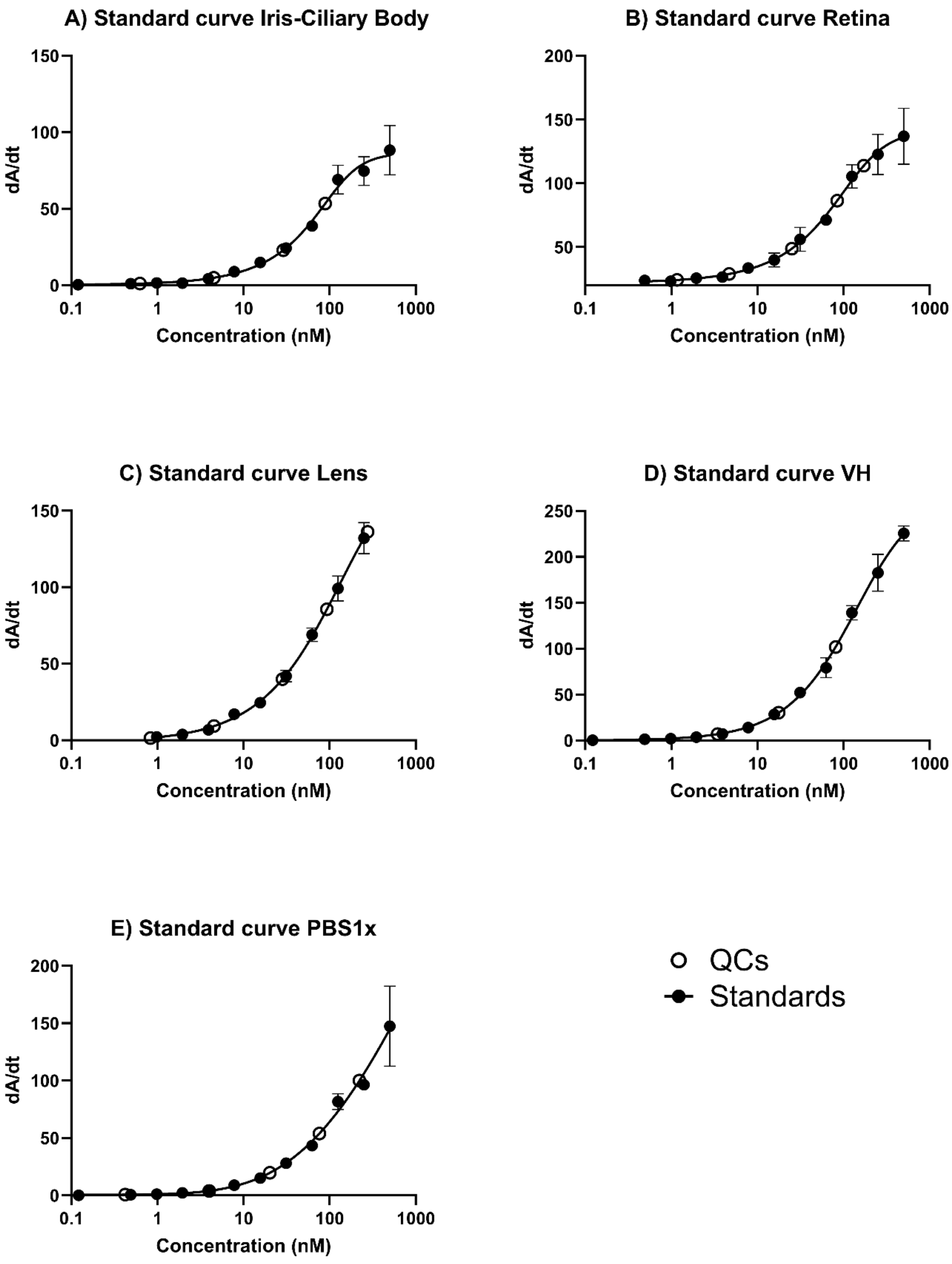

Supplementary Figure S4: Standard curves and QCs for Trastuzumab.

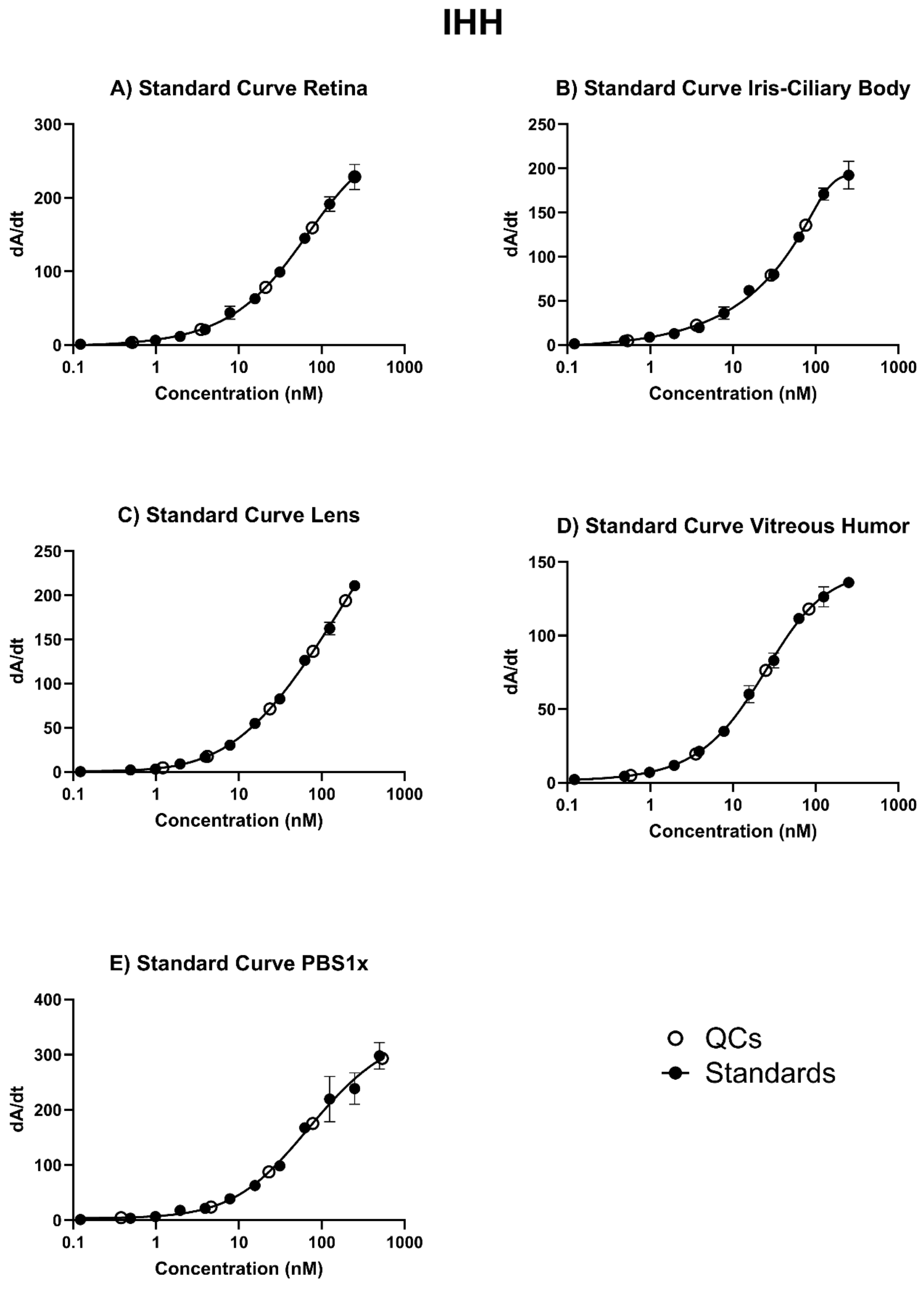

Supplementary Figure S5: Standard curve and QCs of FcRn non-binding trastuzumab (IHH).

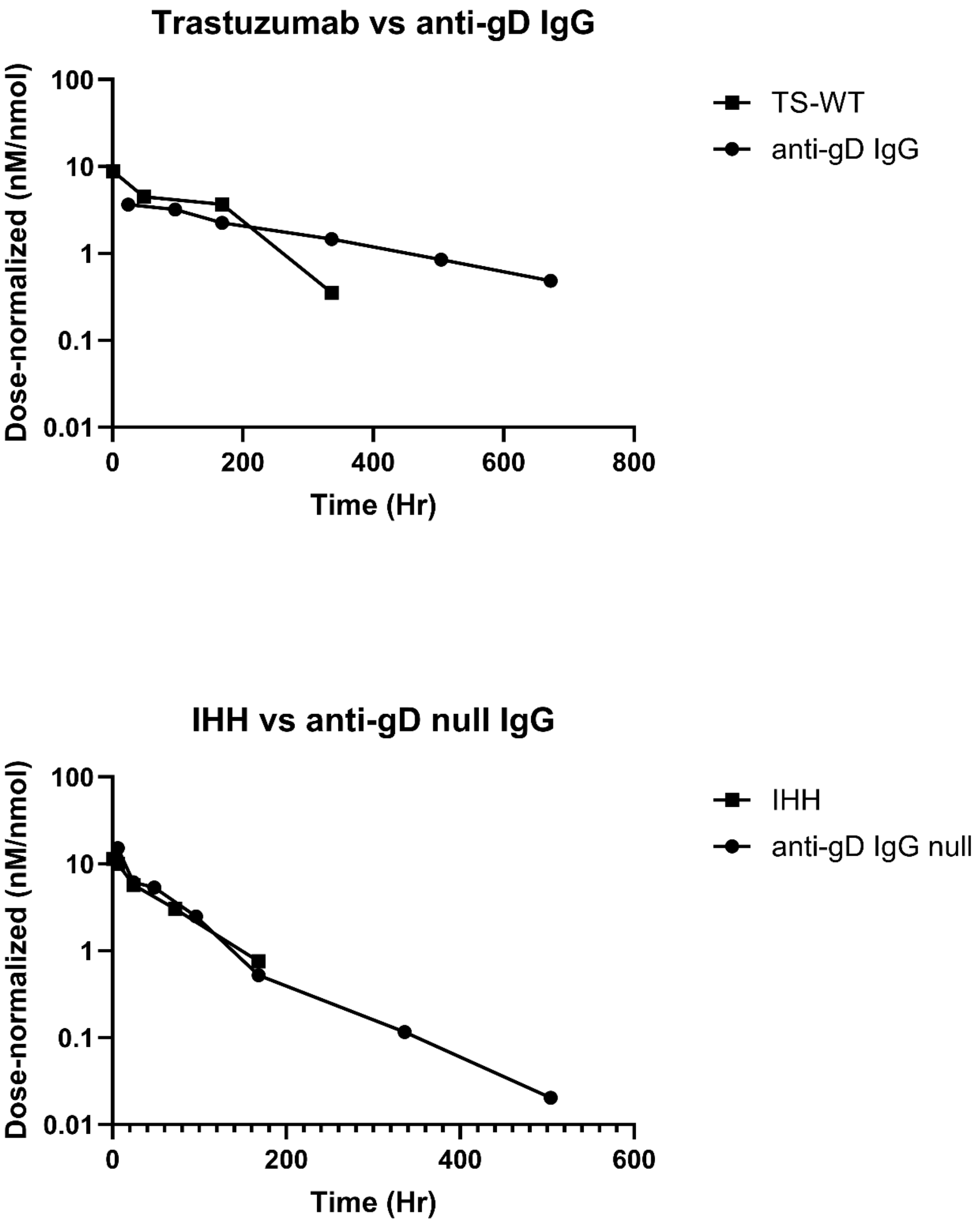

Supplementary Figure S6: Dose-normalized comparison of plasma PK profiles of trastuzumab and its FcRn non-binding variant (IHH) to literature-reported anti-gD IgG (non-targeting) and IgG null (non-FcRn binding) plasma PK profiles.

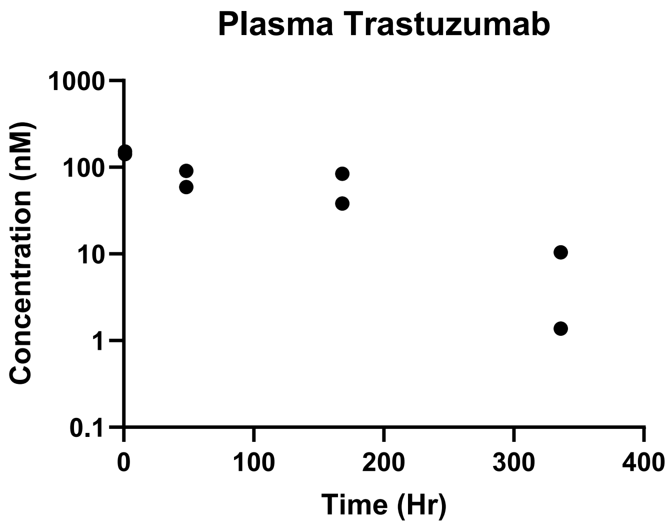

Supplementary Figure S7: Individual-animal plasma concentration–time profiles of wild-type trastuzumab (TS-WT) following intravenous administration (1 mg/kg) in New Zealand White rabbits. Each symbol represents data from an individual animal (n = 2 per time point). Plasma concentrations followed expected IgG kinetics up to 168 h, but both animals showed a pronounced decline at 336 h, deviating from typical IgG elimination behavior. This late-time deviation may reflect biological processes such as the potential development of anti-drug antibodies (ADAs).

Supplementary Table S1: Ocular Tissue Homogenization Methods.

Tissue	Dilution Factor (Reagent)	Homogenization Method
Retina	40x (RIPA-PI)	Sonication, 3 pulses of 10s on and 15s off with an amplitude of 50%
Iris-Ciliary Body	40x (RIPA-PI)	Sonication, 3 pulses of 10s on and 15s off with an amplitude of 75%
Vitreous Humor	2x (1% BSA)	Rotator overnight at 4° C, then the samples were centrifuged at 3000 rpm for 10 minutes
Aqueous Humor	No dilution	After both eyes were immediately enucleated, the aqueous humor of each eyeball was withdrawn into a syringe using a 27-gauge needle (AH tapping or paracentesis), centrifuged at 10,000g for 10 mins 4˚C to get rid of any debris
Lens	10x (RIPA-PI)	Sonication, 3 pulses of 10s on and 15s off with an amplitude of 75%
Cornea	20x (RIPA-PI)	20-30 mg of tissue (3*3 mm) cut; homogenized using 3.0 mm zirconium beads, 10 speed for 5 mins in Bullet Blender
Conjunctiva	10x (RIPA-PI)	20-30 mg of tissue cut; 3 pulses of 10s on and 15s off with a sonication amplitude of 50%
Tears	10x (0.1% BSA-PI)	Centrifugation and solvent extraction methods detailed in Figure 2
